# The science of violence prevention

**DOI:** 10.2471/BLT.15.031015

**Published:** 2015-10-01

## Abstract

Twenty years after Rodrigo Guerrero-Velasco treated violence like a disease, using epidemiology to find the causes, his approach to violence prevention has been taken up across the Americas. He talks to Alyssa Greenhouse.

Q: What were the key health and social problems the city of Cali faced when you were elected mayor in 1992?

A: When I first came to office, communicable and other chronic diseases were no longer the main health issues. Homicide had become the leading cause of death. Violence and insecurity were considered the most serious problems in our city.

Q: You pledged to reduce violence and improve public safety in your 1992 election campaign. After you were elected and unveiled your plans, how were they received?

A: At the time, people thought nothing could be done about the violence and insecurity, but I was convinced that the epidemiological method could be applied to the investigation of a social condition – in this case violence – to help understand its risk factors, just as this method is used in the investigation of the spread of disease. My proposals to remove risk factors, such as restricting the carrying of firearms and the opening hours of bars and discotheques were not at all popular, while my proposals for youth and other social programmes received enthusiastic support.

“People thought nothing could be done about the violence and insecurity.”

Q: Later, you wrote that people were under the misconception that drug cartels and poverty were the main causes of the violence in Cali. Why?

A: It was a natural assumption. The sudden increase in homicides in Colombia in the late 1980s coincided with an increase in the production of cocaine and marijuana. Drug traffickers developed a strong presence in many Colombian cities with displays of money and boastful behaviour. Medellín and Cali were particularly affected and so people attributed the epidemic of homicides to drug trafficking.

Q: So how did you tackle the violence?

A: We knew we had to be clear about the causes of the violence and so we took an epidemiological approach. This approach requires a clear definition of the problem, so we adopted the World Health Organization’s definition of violence that includes the use of force with intention to do harm. We then explored what we call “descriptive variables”, such as the age of victims and perpetrators, their blood levels of drugs and alcohol, possession of firearms, place of occurrence, etc. A prerequisite for this method is reliable information, but much of our information was conflicting. For example, the judiciary reported twice as many homicides as the police. After careful analysis we found that the police only counted the dead in the street – not in hospitals – while the judiciary counted everyone who died in Cali, regardless of where the incidents occurred. To resolve discrepancies and obtain reliable and timely crime data, I called a weekly meeting of representatives from the police, the judiciary and the municipal statistical agency as well as forensic experts and health and social scientists from the city’s university. We called it a “crime observatory”.

Q: What did you find?

A: We found that about 80% of homicides were committed with firearms and that most homicides occurred at weekends or on celebration days, such as Christmas Day and New Year’s Eve, when people consume lots of alcohol. Most of the perpetrators and victims were young men from deprived areas. This epidemiological pattern pointed to social disruption as a major cause, not organized crime. Later we concluded that drug trafficking was similar to the human immunodeficiency virus (HIV) that attacks the body’s defences. People with HIV are susceptible to otherwise innocuous diseases. Similarly, drug traffickers undermined the police and judiciary by offering them bribes and, as a result, society’s defence mechanisms became weak and dysfunctional. Thus people got killed in street fights, in quarrels between neighbours and in arguments over traffic accidents. Contrary to popular belief, we found that drug trafficking was not the main direct cause of the increase in homicides, but rather our law and order institutions were weakened by the drug traffickers.

Q: Did the results of your analysis change perceptions of violence?

A: Yes, in fact, we developed a theoretical model of urban violence by reviewing the literature on the topic and also from our observations of violence in Cali. We found substantial evidence to show that the possession of firearms, the consumption of alcohol and social inequalities – as opposed to poverty per se – were the factors most strongly associated with violent behaviour in Colombia. Other risk factors included a history of child abuse – predisposing some people to violent behaviour – exposure to violent films, and the presence of organized crime.

Q: What solutions did you come up with in Cali?

A: We launched the DESEPAZ programme in 1992, this stands for DEsarrollo, SEguridad, Paz (development, security and peace), a multi-sectoral approach that included social and economic programmes and gun and alcohol restrictions. 

Q: What kind of social and economic programmes did you launch?

A: During my first term the city organized the Casas de Jóvenes (youth clubs) where young people can gather to meet and discuss different issues with an experienced social worker on site. Young people were offered training, for example, in making cobblestones, and the city hired them to pave the streets. We started providing positive parenting courses to prevent child abuse and programmes to prevent bullying in schools. During my second term we built 17 day-care facilities each with about 300 places, some in collaboration with the private sector. All were staffed with nutritionists, psychologists and social workers. I hope that future administrations continue this important work. Finally, we have invested considerably in improving the police and the judiciary and bringing them closer to the people, and we are opening a third Casa de Paz – we opened the first two in my first term – where police, forensic experts and the judiciary work in the middle of Cali’s impoverished neighbourhoods.

Q: How did you continue this work after leaving office in 1994?

A: I helped the Pan-American Health Organization start its first violence-prevention programme in the Americas. It was an opportunity to apply all the lessons we learned in Cali: the application of the public health approach with clear definitions, reliable information, identification of risk factors, planned interventions and evaluation. I also helped to establish the Inter-American Coalition for the Prevention of Violence which is working to develop violence-prevention programmes in 18 countries in the Americas.

Q: How successfully has the Cali approach to violence prevention been applied elsewhere?

A: The epidemiological approach to reducing violence is passing the test in other cities in Colombia and across the Americas. A key part of this approach is to set up crime observatories; regular meetings of all the agencies dealing with crime to gather and analyse data, plan violence-prevention interventions and evaluate them. The Inter-American Development Bank (IDB), the United States Agency for International Development and the World Bank, among others, now recommend creating observatories as a prerequisite for violence-prevention programmes and such observatories have been established in 24 countries in the region. 

Q: What happened in Cali after you left office?

A: Sadly, not all of my successors continued to use a data-driven approach to curbing violence, and only sporadically kept up the youth programmes or the firearm and alcohol policies. The incidence of homicide dropped from 124 per 100 000 population in 1994 to 86 in 1997 then slowly climbed back up to about 100. In contrast, homicides in the Colombian capital of Bogotá, where the epidemiological method was consistently applied by three administrations from 1995 to 2003, dropped from 80 to 20 per 100 000 over this time. 

Q: When you were re-elected as mayor again in 2011 had things changed?

A: Yes. The Colombian police force had become a highly professional and trustworthy body. The powerful drug cartels had largely been dismantled. But the incidence of homicide in Cali was high – around 80 per 100 000 per year – compared to 22 in Bogotá and 70 in Medellín. When we analysed the data, we found that the proportion of homicides due to interpersonal conflict – such as quarrels, alcohol-fuelled brawling and jealousy – had decreased and the proportion attributable to organized crime had increased. These homicides were premeditated, sometimes involving contract killers, some driven by drug problems. The national government and our municipality decided to respond as we had done in the past. Specialists from the police and the prosecutor’s office worked together to identify gang leaders, collect the evidence, and detain and imprison gang members. In 2014 the incidence of homicides in Cali had dropped to 62 per 100 000 and it continues to fall.

Q: What other solutions have you found?

A: In 2011, the city adopted a massive social investment programme benefiting around 600 000 people from the 11 most impoverished districts, focusing on health, education, income generation and food supplements. Total investment in the programme, including support for the police and judiciary, was US$ 40 million in 2014 and in 2015. In 2014, the number of homicides fell by 21% (402) in Cali compared with 2013 (1959) and in the first half of 2015, we had the lowest numbers of homicides in the past 10 years.

Q: Have you ever felt that your own life was at risk?

A: I always stress that in Colombia our drug policies are decided and implemented by the national government. My role is limited to securing compliance with city laws. That may have helped because I have never felt particularly at risk.

Q: What have you learned from your time in office?

A: First, urban violence is preventable. Second, violence prevention requires reliable and timely information. Third, the public health method is a practical approach that can be adapted locally. There is no one-size-fits-all. Fourth, addressing some risk factors associated with violent behaviour, such as reducing social inequalities or establishing healthy child-raising practices, requires time, patience and resources. Addressing other risk factors such as restrictions on alcohol sales or on carrying firearms can be done with fewer resources and achieve rapid results. Finally, violence prevention requires political will on the part of mayors and local officials. 

